# Efficacy of smartphone-based Mobile learning versus lecture-based learning for instruction of Cephalometric landmark identification

**DOI:** 10.1186/s12909-020-02201-6

**Published:** 2020-08-31

**Authors:** Amin Golshah, Fatemeh Dehdar, Mohammad Moslem Imani, Nafiseh Nikkerdar

**Affiliations:** 1 Department of Orthodontics, School of Dentistry, Kermanshah University of Medical Sciences, Kermanshah, Iran; 2 Faculty of Dentistry, Kermanshah University of Medical Sciences, Kermanshah, Iran; 3 Department of Oral and Maxillofacial Radiology, School of Dentistry, Kermanshah University of Medical Sciences, Kermanshah, Iran

**Keywords:** Lateral cephalometry, Smartphone instruction, Lecture-based instruction

## Abstract

**Background:**

Considering the increasing popularity of electronic learning, particularly smartphone-based mobile learning, and its reportedly optimal efficacy for instruction of complicated topics, this study aimed to compare the efficacy of smartphone-based mobile learning versus lecture-based learning for instruction of cephalometric landmark identification.

**Methods:**

This quasi-experimental interventional study evaluated 53 dental students (4th year) in two groups of intervention (*n* = 27; smartphone instruction using an application) and control (*n* = 26, traditional lecture-based instruction). Two weeks after the instructions, dental students were asked to identify four landmarks namely the posterior nasal spine (PNS), orbitale (Or), articulare (Ar) and gonion (Go) on lateral cephalograms. The mean coordinates of each landmark identified by orthodontists served as the reference point, and the mean distance from each identified point to the reference point was reported as the mean consistency while the standard deviation of this mean was reported as the precision of measurement. Data were analyzed using SPSS version 18 via independent sample t-test.

**Results:**

No significant difference was noted between the two groups in identification of PNS, Ar or Go (*P* > 0.05). However, the mean error rate in identification of Or was significantly lower in smartphone group compared with the traditional learning group (*P* = 0.020).

**Conclusions:**

Smartphone-based mobile learning had a comparable, and even slightly superior, efficacy to lecture-based learning for instruction of cephalometric landmark identification, and may be considered, at least as an adjunct, to enhance the instruction of complicated topics.

**Trial registration number:**

This is not a human subject research. https://ethics.research.ac.ir/ProposalCertificateEn.php?id=33714&Print=true&NoPrintHeader=true&NoPrintFooter=true&NoPrintPageBorder=true&LetterPrint=true.

## Background

Effective instruction is the most important factor in academic progression and efficient learning of students. However, the method of instruction should be flexible to match the learning needs of individuals. Cooperation and interaction between students are also important in comprehending the educational content [1–3].

Recent advances in technology have revolutionized the methods of instruction at all levels. Dental education is no exception to this rule [4]. With the advances in computer science, and the availability of high-speed Internet and smartphones, many universities worldwide have debuted long-distance web-based learning or multi-media instruction programs [5]. Electronic learning is growing fast worldwide, and is gaining increasing popularity among learners and mentors. Electronic learning can serve as an effective alternative to traditional education, and improve the quality and quantity of educational medical and dental programs [6–8]. Nkenke et al. [9] showed that technologically-enhanced learning in a theoretical radiological science course decreased the need for lecture-based instruction without negatively affecting the examination results. According to a systematic review, electronic learning has equal effectiveness as traditional classroom learning in enhancement of the knowledge and improvement of clinical performance of students in oral radiology [10].

Mobile learning is defined as “learning across multiple contexts, through social and content interactions, using personal electronic devices” [11]. Of different tools used for electronic learning, smartphones are highly popular among learners due to unique properties such as being user friendly, small size, the ability to connect to the Internet, and easy use [12–14]. Thus, they are well suitable for instruction [15]. Smartphone-based mobile learning should be considered in the context of the literature on mobile learning [16]. Application of smartphones for educational purposes has been considered since they first became available, and they are increasingly used by medical students as a learning aid for several medical applications [16, 17]. In fact, use of smartphone-based applications (apps) as a type of mobile learning has greatly advanced in the recent years and they are extensively used as novel techniques of instruction and learning [17–19].

Lateral cephalometry is an important diagnostic imaging modality for detection of dentoalveolar disorders in orthodontics and orthognathic surgery. Cephalometric assessment involves landmark identification and linear and angular measurements, and is used to determine the morphology of maxillofacial structures and also for treatment planning and assessment of treatment outcome. Moreover, it is essential for diagnosis and treatment of skeletal deformities [20, 21]. Consecutive lateral cephalograms are often requested to study and predict the growth pattern, progression of orthodontic treatment, and prognosis of treatment of orofacial deformities. They are also used to assess the changes caused by the intervention and evaluate the efficacy of treatment [22, 23]. However, lateral cephalometry has some drawbacks as well. For example, it has a number of internal and external confounding factors. The majority of cephalometric measurements require landmark identification and angular and linear measurements, which require high expertise [24]. Moreover, it provides a two-dimensional image of three-dimensional structures; thus, some data are lost and it requires considerable spatial visualization [25]. Therefore, dental students should acquire high proficiency in order to be able to correctly interpret lateral cephalograms [26–28]. Thus, provision of 3D images and videos can greatly help in easier and more efficient learning of this topic [21]. Moreover, considering the significance of accurate landmark identification for more precise interpretation of lateral cephalograms and subsequently more accurate orthodontic treatment planning, it is important to find a technique for more effective instruction of this topic. At present, cephalometric landmark identification is instructed to dental students via traditional lecture-based instruction. Thus, there is an obvious need to employ more advanced technologies for enhanced instruction of this topic.

Considering the gap of information regarding the efficacy of smartphone-based mobile learning as an educational aid in orthodontics [29], this study aimed to compare the efficacy of traditional learning versus smartphone-based mobile learning for instruction of cephalometric landmark identification to dental students.

## Methods

This quasi-experimental, single-blind study evaluated 4th year volunteer dental students of School of Dentistry of Kermanshah University of Medical Sciences in 2018–2019. The students had passed the theoretical course of lateral cephalometry and were selected using census sampling. This study was conducted during the second semester of the 2018–2019 academic year and introduced the use of a mobile application to compare the efficacy of traditional learning versus smartphone-based mobile learning for instruction of cephalometric landmark identification to dental students. Sample size was calculated to be a minimum of 26 students in each group according to a study by Silveira et al., [30], standard deviation of knowledge score to be 2.87 and 2.61 in the traditional learning and smartphone learning groups, respectively, accuracy (d) of 2.5, alpha = 0.05, and power of 90%.

The study was approved by the ethics committee of Kermanshah University of Medical Sciences (IR.KUMS.REC.1397.791). The students were ensured about the confidentiality of their information, and signed informed consent forms prior to participation in the study. All the volunteers were enrolled in the study except for those who reported previous knowledge/training in lateral cephalometric landmark identification. The volunteers were informed that evaluation of their performance in this study would have no effect on their final grade in this course. The study was conducted in the Orthodontics Department by an expert orthodontist. All participants received basic theoretical training regarding lateral cephalometric analysis. The educational program consisted of 2 h of lecture-based learning that focused on theoretical learning regarding lateral cephalometric analysis. The students were then randomly divided into two groups of intervention (smartphone-based mobile learning) and control (lecture-based learning). The educational contents were the same in both groups and focused on tracing of lateral cephalograms and landmark identification.

Intervention group (*n* = 27): Dental students in the intervention group were provided with a smartphone application. This mobile app was designed for the Android operating system to educate the students regarding the identification of landmarks on lateral cephalograms according to the chapter 4 of the Radiographic Cephalometry: From Basics to 3-D Imaging, 2nd edition, by Alexander Jacobson and Richard L. Jacobson in 2007 [23]. The app included seven main topics for cephalometric tracing namely (I) general considerations, (II) marking the soft tissue profile, and outlining the external border of the skull and vertebrae with three subtopics of outlining the soft tissue profile, outlining the external border of the skull in the anterior region from the frontal to the nasal bone, and in the posterior region in the occipital bone, and outlining the first and second cervical vertebrae (C1 and C2), (III) outlining the base of the skull, internal margin of the skull, frontal sinus, and porion, with subtopics of outlining the base of the skull, roof of the orbit, sella turcica, sphenoidal platform, frontal sinus, dorsum sellae, the foramen magnum, the floor of the middle crania fossa and porion, (IV) outlining the maxilla and its related structures in subtopics of nasal bone, piriform foramen, infra-orbital ridge, key ridge, pterygomaxillary groove, anterior nasal spine, nasal floor, posterior nasal spine, maxillary first molars, maxillary anterior region, and maxillary incisors, (V) outlining the mandible with the subtopics of anterior symphysis, symphyseal bone marrow, inferior border of the mandible, posterior ramus, condyles, coronoid and sigmoid notch, anterior ramus, mandibular first molars, and mandibular incisors, (VI) identification of cephalometric landmarks with the subtopics of ANS, Ar, Ba, Bo, Gn, Me, N, Or, PNS, Pog, Po, point A, point B, PTM and S, and (VII) drawing the anatomical planes.

Each topic was clickable to access the subtopics. By clicking on each subtopic, an educational video clip recorded by a skillful instructor (orthodontist) would be played with audio explanations of the practical steps in cephalometric analysis. Written explanations would also appear in a side bar. The user could choose to view the entire text by selecting the continuous scroll. This classification in presentation of topics was intended to enhance the access of users to different topics and accelerate their learning process. One of the authors (F. D.) personally installed the app in the smartphones of students and provided them with the necessary instructions on how to use and navigate it. During the practice time of cephalometric tracing, the mentors supervised the students working with the app and using the pamphlet. The two groups were scheduled for practice at different days. Also, they were strictly requested not to share the educational contents with the other group.

Control group (*n* = 26): Dental students in the control group received the same educational content as did the intervention group in a classroom setting in the form of a 2-h workshop and also received a pamphlet with the same educational content as in the smartphone application, in order to be able to review the taught topics later.

**Fig. 1:**
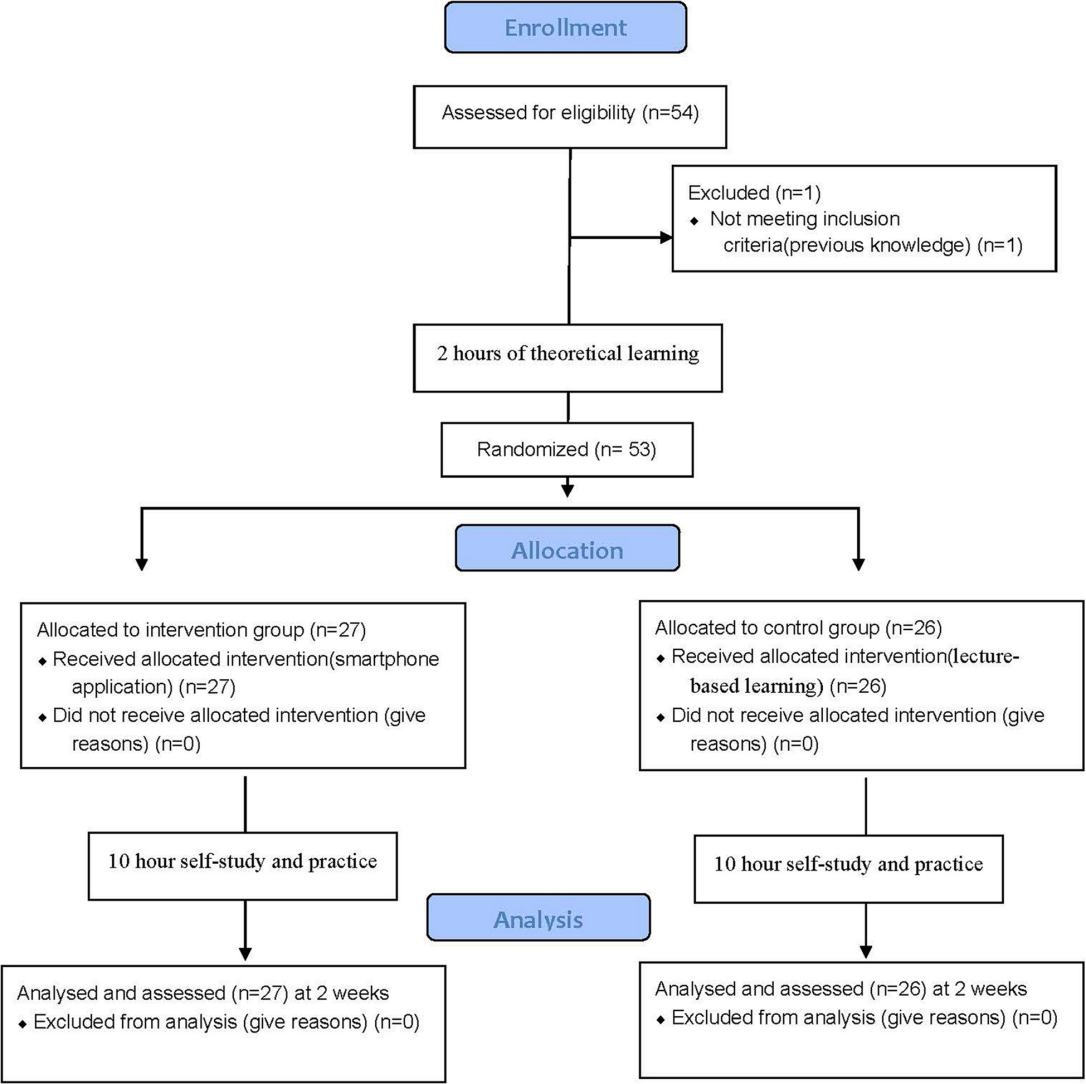
participants’ flow-chart

The students had 10 h of self-study by use of the mobile app or traditional models, depending on their group allocation. Both groups were requested to study the educational contents during their regular attendance in the orthodontics department for 10 h under the supervision of instructors. Two-week time was allowed for the students in the intervention group to use the application and for the students in the control group to review the taught topics and then both groups participated in a test to assess their expertise in cephalometric landmark identification (Fig. 1). Lateral cephalogram of an orthodontic female adult patient with no cleft lip/palate, no supernumeraries, no missing teeth, no anatomical anomalies, no severe asymmetry, no skeletal dysplasia requiring orthognathic surgery, and no use of denture or dental splint was demonstrated to dental students and they were asked to trace the cephalogram and identify four landmarks. The aforementioned four landmarks were selected by two orthodontists and one radiologist in a group discussion and included the posterior nasal spine (PNS), articulare (Ar), gonion (Go) and orbitale (Or).

PNS: The most posterior point on the sagittal plane of the hard palate on the mid-sagittal plane.

Ar: A point at the intersection of the image of the posterior margin of the ramus and the outer margin of the cranial base.

Go: The outer point on either side of the lower jaw at which the jawbone angles upward.

Or: The most inferior point of the inferior border of orbit.

The lateral cephalogram was taken by Cranex 3D (Soredex, Tuusula, Finland) and printed by a laser printer (Dry view 5950; Kodak, USA) using 8 × 10 in. Kodak medical X-ray film.

**Fig. 2:**
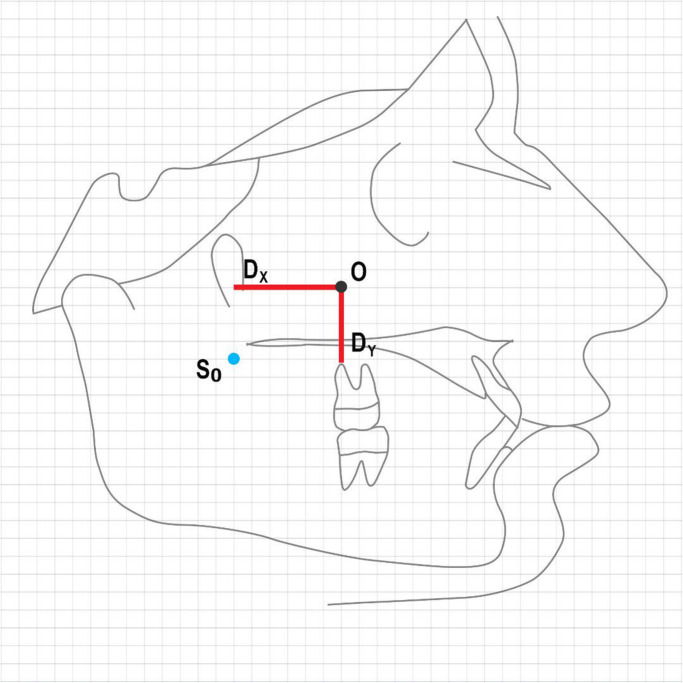
Schematic view of the comparison of landmarks identified by students with the reference points (S0 = Reference point, O = Landmark identified by student, Dx: Distance between the two points in the X axis, Dy: Distance between the two points in the Y axis, O-S0 distance: $D=\sqrt{{\mathrm{Dx}}^2+{\mathrm{Dy}}^2}$)

The X and Y coordinates of each landmark identified by dental students were compared with the reference points identified by the orthodontists. The mean distance between the identified landmark and the reference point was calculated and reported as the mean consistency while the standard deviation of this mean was reported as the accuracy of measurement for each group (Fig. 2).

Data were analyzed using SPSS version 18 (SPSS Inc., IL, USA). Normal distribution of data was evaluated using the Kolmogorov-Smirnov test, which showed that the data were normally distributed. Thus, the two groups were compared using independent sample t-test. The Chi-square test was applied to assess the correlation of gender and study group. Level of significance was set at 0.05.

## Results

A total of 53 dental students participated in this study; out of which, 27 (50.9%) were males and 26 (49.1%) were females. A total of 27 dental students were evaluated in the smartphone-based mobile learning group including 13 males (48.1%) and 14 females (51.9%). Also, 26 dental students were evaluated in the traditional learning group including 14 males (53.8%) and 12 females (46.2%). The two groups were not significantly different in terms of gender (Chi-square test, *P* = 0.678). The mean age of students was 22 ± 1.63 years.

The mean grade point average of students was 15.39 ± 1.09 in the traditional learning and 15.57 ± 0.91 in the smartphone-based mobile learning group. The difference in this regard was not significant between the two groups (Independent sample t-test; *P* = 0.503).

Table 1 compares the two groups regarding errors in identification of the chosen cephalometric landmarks. According to independent sample t-test, the two groups were not significantly different in identification of PNS (*P* = 0.960), Ar (*P* = 0.467) or Go (*P* = 0.120). However, the mean error in identification of Or was significantly lower in the smartphone group (*P* = 0.020).

**Table 1:** Comparison of the two groups regarding errors in identification of the chosen cephalometric landmarks

Point	Group	Mean	Std. deviation	Minimum	Maximum	*P* value
PNS	Traditional learning	4.85	3.15	0	11	0.960
	Smartphone-based mobile learning	4.89	2.95	0	10	
Ar	Traditional learning	21.65	6.75	2	39	0.467
	Smartphone-based mobile learning	20.52	4.32	13	32	
Go	Traditional learning	6.92	3.72	2	19	0.120
	Smartphone-based mobile learning	5.56	2.47	2	12	
Or	Traditional learning	6.65	6.24	2	27	0.020
	Smartphone-based mobile learning	3.67	1.64	0	7	

## Discussion

This study compared the efficacy of smartphone-based mobile learning versus lecture-based learning for cephalometric landmark identification by dental students. Dental students in the two groups were matched in terms of age, gender and grade point average, which was in agreement with the methodology of some previous studies [31, 32]. This was done to eliminate the confounding effect of these variables on the results. No significant difference was noted between the two groups in identification of PNS, Ar or Go (*P* > 0.05). However, the mean error rate in identification of Or was significantly lower in the smartphone group (*P* = 0.020). It appears that identification of Or is somehow difficult due to the superimposition of anatomical structures in the orbit. Thus, the error rate in identification of Or was higher than that for the three other points (which are easier to identify). The landmark identification error for the Or has been previously evaluated by Major et al. [33]. According to them, this point has a significant identification error in the Y axis (vertical dimension). Also, another study evaluated the reliability of anatomical landmark identification for different radiographic points and introduced the Or as a point with greater intraobserver variations in two-dimensional cephalometric analysis; whereas, PNS and Go had lower rate of variations in identification [34]. They also added that in two-dimensional assessment, the observers often try to identify the landmarks based on the adjacent structures, or distinguishing the contrast between the radiopaque and radiolucent structures. For instance, most observers were not capable of precisely repeating their identification of Or in the x-direction for the second time. This is probably due to the superimposition of images of the left and right orbits, and their more horizontal rather than vertical alignment. It is assumed that the electronic method has greater efficacy for improvement of the performance of students with regard to identification of Or (which is harder to detect than other points) since this method allows for easier access to the educational content, and the users can repeatedly watch the related videos to master it.

In a similar study in Basel University in Switzerland, the results showed that students in electronic learning group experienced 10% improvement in their level of knowledge compared with those in the traditional learning group [35]. This finding was in agreement with our results regarding knowledge enhancement in the smartphone-based mobile learning group. Mitchell et al. [36] evaluated 231 nursing students and reported that students who had continuous access to educational content electronically gained higher scores. Basoglu and Akdemir [37] also confirmed the positive effect of using educational applications on the academic progression and learning of students, which was in agreement with our findings. Fernandez-Lao et al. [38] reported optimal efficacy of a smartphone application for enhancement of skills regarding ultrasound imaging. Leasure et al. [39] showed that electronic learning was 19% more effective than the traditional learning. Fozdar and Kumar [40] and Hartnell-Young and Heym [41] showed that educational smartphone applications had optimal efficacy for enhancement of learning.

Studies on the efficacy of electronic learning for instruction of cephalometric landmark identification are limited. Silveira et al., [30] in a randomized clinical trial in Brazil evaluated the learning process of lateral cephalometry by dental students using a learning virtual object, and found that it was an efficient and effective tool for enhancement of the learning process and can greatly help in instruction of cephalometry. Their results were in agreement with our findings.

In contrast, some other studies have stated that adequate conditions are not available for replacement of traditional learning with electronic learning and these two methods of instruction should be preferably used in combination with each other [42]. For instance, Kavadella et al., [43] in Greece evaluated the efficacy of traditional instruction combined with electronic instruction compared with traditional instruction alone for oral and maxillofacial radiology topics and showed that the performance of the group that received combined instruction was significantly superior to the performance of the traditional learning group. Meckfessel et al., [44] at the School of Dentistry of Hannover, Germany demonstrated the superior efficacy of the combined instruction technique to the traditional instruction alone. Sendra-Portero et al. [45] stated that electronic learning can be used as an alternative to traditional learning for instruction of radiology topics to dental students with no adverse effect on the learning process. However, they added that interaction and communication of students with their mentors and their attendance to classes are also an important part of the learning process. Thus, they suggested electronic instruction at first followed by holding several classes for in-person problem solving. A recent study reported enhanced medical education and exam performance following tablet computer-based integrated training and clinical practice [46].

Most previous studies used electronic learning as an adjunct to traditional learning and showed its relative superiority compared with the traditional learning alone. In the present study, electronic learning alone was compared with traditional learning alone, and the results revealed that electronic learning was comparable (or even slightly superior) to the traditional method in identification of one landmark. The efficacy of the two methods of learning was the same in identification of the other three landmarks. Thus, it may be concluded that smartphone-based mobile learning may be able to enhance the process of learning, at least for some topics, since it is popular, easily accessible and effective. Smartphone-based mobile learning has high potential for knowledge promotion and encouraging the students especially when used in combination with traditional learning. Future studies are recommended to design applications for instruction of other topics to dental students.

This study had some limitations such as small sample size. Future studies with larger sample size and in other universities are required to further assess the efficacy of electronic learning in instruction of complex topics.

## Conclusion

Smartphone-based mobile learning had a comparable, and even slightly superior, efficacy to lecture-based learning for instruction of cephalometric landmark identification. Thus, it may be considered, at least as an adjunct, to enhance the instruction of complicated topics.

## Data Availability

All materials described in this manuscript including all relevant raw data, will be freely available to any scientist wishing to use them for non-commercial purposes, without breaching participant confidentiality.

## Abbreviations

PNS: Posterior Nasal Spine
Or: Orbitale
Ar: Articulare
Go: Gonion
